# How to follow the new EU Council recommendation and improve prostate cancer early detection: the Prostaforum 2022 declaration

**DOI:** 10.1016/j.euros.2023.05.011

**Published:** 2023-06-08

**Authors:** Ondřej Májek, Marek Babjuk, Monique J. Roobol, Ola Bratt, Hendrik Van Poppel, Roman Zachoval, Jiří Ferda, Marcela Koudelková, Ondřej Ngo, Jakub Gregor, Sarah Collen, Karel Hejduk, Ladislav Dušek, Vlastimil Válek

**Affiliations:** a National Screening Centre, Institute of Health Information and Statistics of the Czech Republic, Prague, Czechia; b Faculty of Medicine, Masaryk University, Brno, Czechia; c Department of Urology, 2nd Faculty of Medicine, Charles University, Motol University Hospital, Prague, Czechia; d Department of Urology, Erasmus University Medical Centre, Cancer Institute, Rotterdam, The Netherlands; e Department of Urology, Institute of Clinical Sciences, Sahlgrenska Academy, University of Gothenburg, Gothenburg, Sweden; f Department of Urology, Sahlgrenska University Hospital, Gothenburg, Sweden; g European Association of Urology Policy Office, Arnhem, The Netherlands; h Department of Urology, KU Leuven, Leuven, Belgium; i Department of Urology, 3rd Faculty of Medicine of Charles University and Faculty Thomayer Hospital, Prague, Czechia; j Department of Imaging Methods, Medical Faculty Pilsen, Charles University, University Hospital Pilsen, Pilsen, Czechia; k Ministry of Health of the Czech Republic, Prague, Czechia

**Keywords:** Cancer screening, Pilot studies, Prostate cancer

## Abstract

An updated Council of the EU recommendation on cancer screening was adopted in December 2022 during the Czech EU presidency. The recommendation included prostate cancer as a suitable target disease for organised screening, and invited countries to proceed with piloting and further research. To support further discussions and actions to promote early detection of prostate cancer, an international conference in November 2022 (Prostaforum 2022) resulted in a joint declaration. Here we describe the EU policy background, summarise the preparation of the declaration and the key underlying evidence and expert recommendations, and report the text of the declaration. The declaration summarises the striking inequalities in prostate cancer burden in Europe and calls on all stakeholders to consider and support concrete steps for advancement of organised early detection of prostate cancer. Our aim is to request endorsement of the text and potential initiation of practical actions by all stakeholders to support the aims of the declaration.

**Patient summary:**

Prostate cancer is among the most frequent cancers and is one of the most common causes of cancer death among men. The European Union has recommended new pilot programmes for prostate cancer screening. The Prostaforum 2022 declaration invites all stakeholders to support this new recommendation with specific steps.

Cancer control was one of the key priorities of the recently concluded Czech presidency of the Council of the EU. In December 2022, the updated council recommendation on cancer screening was adopted [1] following a proposal by the European Commission and negotiations in the Council of the EU. The recommendation includes prostate cancer as a suitable target disease for organised cancer screening. While acknowledging that further implementation and outcomes research is still needed to evaluate and optimise the feasibility, effectiveness, and cost-effectiveness in real-life practice, the recommendation encourages countries to progress with practical implementation in light of the scientific opinion of the Group of Chief Scientific Advisors [2], which states that there is good evidence that screening can reduce mortality from prostate cancer.

In November 2022, the international Prostaforum conference [3] was organised to discuss the state of knowledge and further steps to harness the potential of organised prostate cancer screening in Europe. The conference utilised the expertise of an international consortium of experts on prostate cancer screening who had come together on an EU4Health co-funded project to monitor and strengthen the implementation of innovative approaches for prostate cancer screening, called PRAISE-U (grant number 101101217, starting on April 1, 2023).

Conference speakers presented underlying evidence and practical experience with organised testing; presentations are available at the conference website [3]. Unorganised prostate-specific antigen (PSA) testing, which is considered ineffective, is already widespread. By contrast, organised PSA screening programmes can reduce the individual risk of dying from prostate cancer by up to approximately 50% [4]. Overdiagnosis of clinically insignificant prostate cancers can be reduced via individual risk stratification and magnetic resonance imaging (MRI) [5]. The new evidence led the European Association of Urology (EAU) to recommend a risk-adapted strategy for early detection of prostate cancer [6] and a call for endorsement by the European Commission [7]. Nevertheless, the risk-adapted screening strategy has its challenges, including a need for appropriate infrastructure, training, and quality assurance in different health care systems with differing resource availability [8]. Organised testing is a complex, resource-intensive endeavour requiring an administrative system and a registry platform, as demonstrated by the Swedish prostate cancer testing programme [9], and several algorithm-related knowledge gaps remain (eg, compliance of participants and health care professionals, optimal indications for MRI). Appropriate management of localised prostate cancer with avoidance of overtreatment still represents a particular challenge [10].

To support further policy discussions and actions to promote early detection of prostate cancer across the EU, a declaration with a call for policy actions at the national and EU level was drafted and presented at the Prostaforum conference. The full text of the declaration, as well as a list of individuals and organisations endorsing the declaration and a form for its endorsement, is available at https://prostaforum.uzis.cz/en/prague-prostaforum-declaration/.

## Prostaforum 2022 declaration

During a meeting in September 2022, the Ministers of Health of the European Union expressed their support for the Call to Action on Oncology, which was the main output from the Modern Cancer Control: Saving Lives Through Smart Solutions expert conference on oncology, which called on all stakeholders to consider support of EU-wide accessibility of quality cancer screening programmes [11].

Prostate cancer is the most frequently occurring male cancer in many European countries and one of the most common causes of cancer death among men. Estimated prostate cancer incidence rates varied twofold and mortality rates threefold in 2020 across the EU [12]. Detection of prostate cancer at an early stage, when treatment is less invasive and more likely to be effective, could significantly reduce mortality and the incidence of advanced prostate cancer with serious impacts on quality of life among European men. According to the evidence review report produced by the Science Advice for Policy by European Academies body for the European Commission [2], there is good evidence that prostate cancer screening with PSA testing reduces deaths from prostate cancer. While overdiagnosis and overtreatment are major harms in prostate cancer screening, use of an upper age limit, including risk stratification after baseline PSA, high-quality MRI scanning, and other complementary tests will significantly reduce overdiagnosis and improve the harm-to-benefit ratio.

Taking the above-mentioned considerations into account, the Prostaforum declaration calls on all stakeholders to consider and support the following steps specifically for prostate cancer control through early detection:

• Adopt and implement in a timely and ambitious manner the new council recommendation on cancer screening to promote EU-wide state-of-the-art provision of evidence-based cancer screening.
• Make available in a timely manner new technologies for early detection of prostate cancer.
• Investigate these new technologies in screening algorithms applied to population-based screening programmes.
• The European Commission should promote the development and implementation of European guidelines and quality assurance schemes for prostate cancer screening through a European initiative on prostate cancer, so that countries can adhere to the best evidence-based recommendations, while at the same time recognising their local circumstances and resource constraints, among other factors.
• The European Commission should consider the possibility of supporting collaborative efforts to implement actions to support early detection of prostate cancer at the national level, including health literacy and raising of awareness.
• The European Commission, together with member states with reference to their national context, should make every effort to reduce the striking differences between EU countries in the burden of prostate cancer.
• Structured sharing of good practices in prostate cancer screening and early detection among EU countries, including timely sharing of data on agreed quality assurance indicators and the results of implementation/pilot studies, should be further strengthened.
• Support all stages of research (preclinical → clinical → implementation → outcomes) aimed at improving prostate cancer screening and early detection as measures to decrease the striking differences in prostate cancer mortality between EU member states.

## Concluding remark

We hope that the preparation of the EU Council recommendation following the EAU recommendation and this declaration indicate a new phase of evidence-based EU policy for early detection of prostate cancer. We agree that vast differences in cancer mortality within the EU are partly a reflection of uneven implementation of effective medical practices, including recommended screening programmes [13]. Creative and ambitious implementation of the new council recommendation will be essential in efforts to reduce these differences. We have shared the text of the declaration here and request its endorsement by relevant stakeholders and initiation of practical actions to support its aims.

  ***Author contributions:*** Ondřej Májek had full access to all the data in the study and takes responsibility for the integrity of the data and the accuracy of the data analysis.

  *Study concept and design*: Májek, Babjuk, Roobol, Bratt, Van Poppel, Zachoval.

*Acquisition of data*: Májek, Babjuk, Roobol, Bratt, Van Poppel, Zachoval.

*Analysis and interpretation of data*: Májek, Roobol, Bratt, Van Poppel.

*Drafting of the manuscript*: Májek, Roobol, Bratt, Van Poppel, Collen.

*Critical revision of the manuscript for important intellectual content*: Májek, Babjuk, Roobol, Bratt, Van Poppel, Zachoval, Ferda, Koudelková, Ngo, Gregor, Collen, Hejduk, Dušek, Válek.

*Statistical analysis*: None.

*Obtaining funding*: Májek, Hejduk, Dušek.

*Administrative, technical, or material support*: Koudelková, Ngo, Gregor.

*Supervision*: Májek, Babjuk, Roobol, Bratt, Van Poppel, Zachoval, Dušek, Válek.

*Other*: None.

  ***Financial disclosures:*** Ondřej Májek certifies that all conflicts of interest, including specific financial interests and relationships and affiliations relevant to the subject matter or materials discussed in the manuscript (eg, employment/affiliation, grants or funding, consultancies, honoraria, stock ownership or options, expert testimony, royalties, or patents filed, received, or pending), are the following: None.

  ***Funding/Support and role of the sponsor:*** This work was supported within the framework of the European Social Fund, Operation Programme Employment (National Coordination Centre for Disease Early Detection Programmes, grant no. CZ.03.2.63/0.0/0.0/15_039/0006904). The sponsor played no role in the design and conduct of the study, interpretation of the data, and preparation of the manuscript.
